# Food insecurity among university students in Australia: A systematic review of prevalence, predictors, health and academic outcomes

**DOI:** 10.1111/1747-0080.70082

**Published:** 2026-06-04

**Authors:** Suvasish Das Shuvo, Karen Charlton, Rajshri Roy, Margaret Allman‐Farinelli, Seema Mihrshahi, Katherine Kent

**Affiliations:** ^1^ School of Medical, Indigenous and Health Sciences, Faculty of Science, Medicine and Health University of Wollongong Wollongong New South Wales Australia; ^2^ Department of Nutrition and Food Technology Jashore University of Science and Technology Jashore Bangladesh; ^3^ School of Health Sciences, College of Health, Medicine and Wellbeing The University of Newcastle, University Drive Callaghan New South Wales Australia; ^4^ Nutrition and Dietetics, Susan Wakil School of Nursing and Midwifery, Faculty of Medicine and Health The University of Sydney Sydney New South Wales Australia; ^5^ Charles Perkins Centre The University of Sydney Sydney New South Wales Australia

**Keywords:** diversity, equity, financial stress, higher education, inclusion, systematic review

## Abstract

**Aims:**

Food insecurity has increasingly been recognised as a significant challenge among university student populations in Australia. This systematic review aimed to synthesise existing evidence on the prevalence, predictors, and outcomes of food insecurity among university students in Australia.

**Methods:**

A comprehensive search was conducted (May 2025), following PRISMA guidelines, across eight databases (Scopus, Web of Science, Google Scholar, ERIC, MEDLINE, CINAHL, Informit, and PsycINFO). Studies were included if peer reviewed, published in English since 2000, and food insecurity was assessed among students attending tertiary institutions in Australia. Two authors independently screened search results against the inclusion criteria.

**Results:**

From 1450 records, 23 met the inclusion criteria: 20 quantitative (*n* = 105–3077) and 3 qualitative or mixed methods (*n* = 14–64). 17 studies assessed the prevalence of food insecurity, reporting rates between 9.0% and 77.9%. Fifteen studies examined predictors of food insecurity. Key risk factors included being an international student (*n* = 2), low income/limited financial resources (*n* = 3), unstable employment (*n* = 3), younger age (*n* = 5), and living alone (*n* = 9). 6 studies found associations between food insecurity and poor dietary patterns; 5 studies reported associations with poorer physical health. 6 studies identified associations with increased anxiety, depression, and stress, and 2 of 4 studies reported associations with poorer academic performance. Qualitative findings suggested that food insecurity exacerbated existing health conditions and weight management. Studies evaluating interventions to improve student food insecurity were not identified.

**Conclusion:**

Food insecurity is highly prevalent among Australian university students and was associated with poorer diet and health, and to reduced academic performance. Despite evidence of widespread impact, no evaluated interventions were identified, underscoring the need for coordinated research and policy action in higher education.

## 1 INTRODUCTION

Food insecurity occurs when people have limited or uncertain access to nutritionally adequate, affordable, culturally acceptable, and safe foods, or when they are unable to acquire these foods in socially appropriate ways.^1^, ^2^ Food insecurity is a significant and growing problem among university students both in Australia and globally, with different studies showing that students are at a higher risk than the general population.^3^, ^4^, ^5^ Recent reviews report prevalence to range from 21% to as high as 82%.^3^, ^6^, ^7^ Food‐insecure students are more likely to skip meals and consume fewer healthy foods, highlighting the serious dietary impact of food insecurity in this population.^8^

Multiple, intersecting factors contribute to the high rates of food insecurity in the student population, including limited financial resources, high tuition fees, lower purchasing power, increasing housing costs, and limited access to affordable and healthy food.^9^, ^10^, ^11^ One of the primary drivers is financial strain. Many university students rely on limited sources of income such as casual or part‐time employment, student loans, or family support, all of which may be unstable or insufficient.^12^ The rising cost of living, particularly housing, tuition, and transportation, often forces students to make trade‐offs, with food frequently being the most flexible expense.^13^, ^14^ International students may face additional challenges, including restricted work rights, language barriers, and limited access to social safety nets.^12^, ^14^, ^15^ Furthermore, certain groups such as Indigenous students, students from rural and regional areas, and students with dependents are at increased risk due to structural inequalities and compounding socioeconomic disadvantage.^12^, ^16^ A lack of financial literacy and time to prepare meals may also contribute to the problem.^17^, ^18^

The health and academic consequences of experiencing food insecurity appear to be significant and multifaceted.^11^ Students experiencing food insecurity often consume cheaper, calorie‐dense foods that have low nutrient content, thereby contributing to both malnutrition and obesity.^19^, ^20^ In terms of mental health, the constant uncertainty surrounding food access contributes to heightened levels of chronic stress, anxiety, and depression.^21^, ^22^ These mental health challenges are compounded by physical health effects, including fatigue, weakened immunity, and increased vulnerability to illness.^23^ Ultimately, this may lead to negative impacts on academic performance, including poor concentration, increased absenteeism, and a greater risk of dropping out of university.^24^, ^25^, ^26^, ^27^, ^28^ The ongoing stress of not knowing where the next meal will come from can undermine a student's ability to engage and succeed in their studies.^29^

Recognising these impacts, various interventions have been implemented internationally, and in Australia, to provide short‐term relief to students.^30^, ^31^, ^32^ Australia's higher education sector is increasingly adopting the Health Promoting University model that emphasises the creation of supportive campus environments that actively promote health and well‐being.^33^, ^34^, ^35^ Common strategies include on‐campus food pantries or food hubs, subsidised meal programs, financial aid schemes, food vouchers, community kitchens, and financial literacy workshops.^11^, ^31^, ^36^ In the United States and Canada, institutional and governmental responses have increasingly incorporated student‐centred supports, often informed by comprehensive needs assessments and longitudinal studies. While these initiatives offer important relief, there is increasing recognition that structural or upstream interventions such as comprehensive student support policies and income assistance are needed for sustainable impact.^31^, ^37^

Numerous systematic reviews documenting food insecurity among university students have been conducted, particularly from the United States and Canada. These findings are not directly transferable to Australia due to differences in higher education systems, social policy frameworks, and student support structures.^5^, ^6^, ^38^, ^39^, ^40^ In Australia, income support programs such as Youth Allowance and Austudy, along with universal healthcare, create a distinct policy environment that shapes student experiences of financial stress and access to basic needs, including food.^41^ The Universities Accord Interim Report (2023) underlines the growing concern that financial pressures are becoming a major barrier to accessing and succeeding in higher education in Australia.^30^ It identifies food insecurity as a key concern, particularly among equity groups including students from low socioeconomic backgrounds, regional and remote areas, and Aboriginal and Torres Strait Islander communities who face disproportionate hardship. To support this, empirical research on food insecurity among university students in Australia is growing, yet remains fragmented, with no comprehensive synthesis of national data. As universities increasingly adopt health‐promoting approaches, there is a clear need for a systematic review to consolidate current findings and better understand the impact of food insecurity on student health, diet quality, academic outcomes, and to identify those at greatest risk among the student body. Therefore, this review synthesises existing evidence on the prevalence, associations, and outcomes of food insecurity among Australian university students to inform context‐specific interventions and policy responses.

## 2 METHODS

This systematic review was conducted in accordance with the Preferred Reporting Items for Systematic Reviews and Meta‐Analyses (PRISMA) guidelines.^42^ The completed PRISMA 2020 checklist is provided as Supporting Information. The review protocol was registered with the International Prospective Register of Systematic Reviews (PROSPERO; Registration No. CRD420251033853, Date: 27 April 2025).

Studies were those that included an assessment of food insecurity, including those examining the impact of food insecurity on physical and mental wellbeing among students attending tertiary institutions in Australia. Studies were included that involved post‐secondary students (both domestic and international) enrolled in Australian universities or higher education institutions, including Technical and Further Education and other vocational education providers. Only peer‐reviewed articles published in English from the year 2000 onwards were considered. Studies were excluded if they focused on non‐post‐secondary populations (e.g., primary or secondary school students), general populations without specific data on students, or higher education contexts where students were not distinguished from staff. Opinion pieces, commentaries, unpublished theses, and studies lacking relevant food insecurity data or outcomes such as prevalence, health, academic performance, or coping strategies were also excluded.

A systematic search was conducted across eight electronic databases including Scopus, Web of Science, Google Scholar, ERIC, MEDLINE, CINAHL, Informit, and PsycINFO in May 2025. The search strategy focused on identifying any studies that assessed food insecurity in Australian university students (Table S1 for search terms). Only peer‐reviewed articles published in English were considered for inclusion. All identified articles were imported into Covidence, a web‐based platform for managing systematic reviews.^43^ Duplicate records were removed automatically. Two reviewers independently screened all articles using a three‐stage process. First, titles and abstracts were reviewed against the predefined eligibility criteria. Full‐text articles were then retrieved and independently assessed by at least two reviewers. Any disagreements were resolved through discussion and consensus, with support from a third reviewer as required.

Studies that satisfied the inclusion criteria after the full‐text review proceeded to data extraction. A standardised electronic data extraction form was used to gather information such as the study author, year of data collection, study population, sample size, methods of data collection, measures of food security outcomes, findings specific to university students, and key results. One researcher performed the initial data extraction, while two others independently reviewed all extracted data to ensure accuracy. Any discrepancies were resolved through discussion and consensus. In cases where information was ambiguous or missing, the study's corresponding author was contacted for clarification.

Study quality was appraised using the relevant Joanna Briggs Institute critical appraisal checklist for each study design by two authors independently: cross‐sectional, cohort, and qualitative, ensuring methodological rigour was assessed consistently across all included studies.^44^

A tabular summary was used to present study characteristics, measurement tools, and reported outcomes. Differences between food‐secure and food‐insecure students were extracted, including statistical significance where provided. Due to the heterogeneity of measures, a narrative synthesis was undertaken to collate prevalence estimates and summarise associations with demographic, health, dietary, and academic outcomes.

## 3 RESULTS

**FIGURE 1:**
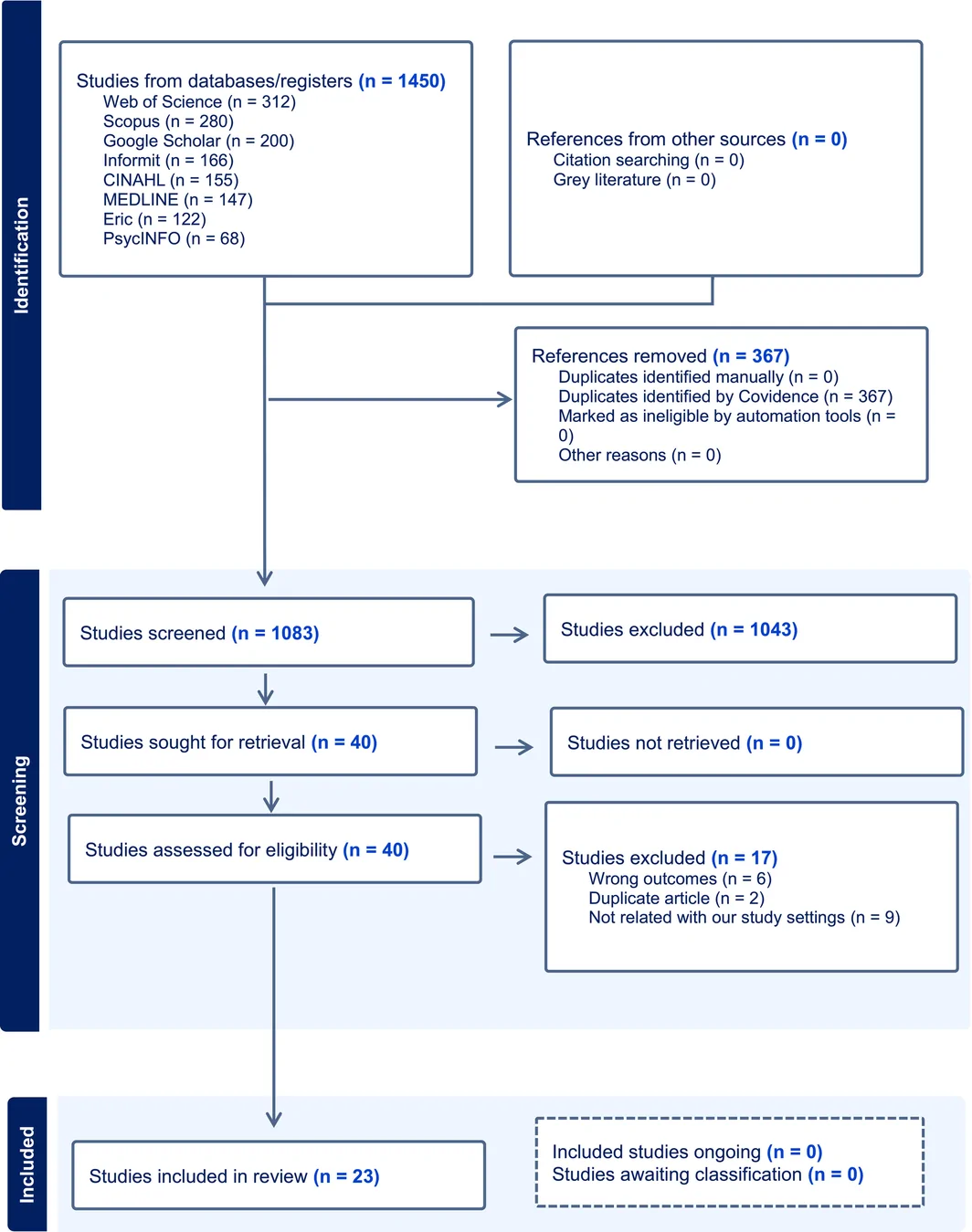
PRISMA flow diagram of search strategy resulting in included studies.

As summarised in the PRISMA diagram (Figure 1), a total of 1450 unique records were retrieved from eight databases. Following the removal of duplicates, 1083 unique articles remained and were subjected to title and abstract screening. From these, 40 articles were selected for full‐text review. Ultimately, 23 articles met the eligibility criteria and were included in the systematic review, whereas one of these studies was conducted in Australia and New Zealand. The remaining articles were excluded at the full‐text stage for the following reasons: 6 studies had wrong outcomes; 2 were duplicate articles; and 9 were not related to our study setting.

Of the included studies, three were conducted at Macquarie University, (*n* = 3),^11^, ^19^, ^22^ University of Tasmania (*n* = 3),^32^, ^45^, ^46^ University of Sydney (*n* = 3),^14^, ^20^, ^47^ and University of Melbourne (*n* = 3)^48^, ^49^, ^50^ respectively. The remaining studies were carried out at the University of Wollongong (*n* = 2),^31^, ^51^ University of Newcastle (*n* = 2),^12^, ^52^ and other institutions in Australia (*n* = 7). Most studies were quantitative (*n* = 20), including cross‐sectional studies (*n* = 19)^9^, ^12^, ^14^, ^15^, ^19^, ^20^, ^21^, ^22^, ^31^, ^32^, ^45^, ^46^, ^50^, ^51^, ^52^, ^53^, ^54^, ^55^, ^56^ and one longitudinal study (*n* = 1).^48^ The sample size of quantitative studies ranged from 105 to 3077.^22^, ^52^ One study employed a qualitative research design,^47^ combining focus groups and semi‐structured interviews,^11^ and one used a mixed‐methods approach.^50^ Sample sizes in qualitative and mixed‐methods studies ranged from 14 to 64.^47^, ^50^ Of the included studies, most (*n* = 21) captured food insecurity‐related data using online surveys (Table 1). Most studies included a range of student types, including undergraduate and postgraduate students and domestic and internationally enrolled students. Three studies included international students only.^9^, ^47^, ^48^, ^50^

**TABLE 1:** Overview of included studies that reported the year, setting, sample size, design and analytical approach in the respective study settings.

Author, year, and references	Data collection time frame	Setting	Sample size and demographic characteristics	Study design	Analytical approach
Bennett et al. (2022)^9^	Five time points across 2020 (May, July, August, October and December).	Monash University, Victoria, Australia, at its Australian (multi‐site) or Malaysian campuses.	*n* = 1315 students were included.	Cross‐sectional analysis and online survey.	Multivariable regression analysis and a sensitivity analysis.
Brownfield et al. (2023)^15^	Not mentioned	Swinburne University of Technology, Melbourne, Australia	*n* = 664 university students (56.5% female, 91.7% domestic students)	Cross‐sectional online survey	Regression and mediation analysis.
Waite et al. (2025)^50^	March and July 2022	Participants had to have been living in Australia for at least 3 months, have been studying at a Melbourne‐based tertiary institution and be on a temporary visa that allowed them to study in Australia.	*n* = 64 individuals (68.5% female, 44.5% aged 25 years or less)	Mixed methods study (online survey, semi‐structured interview)	Chi‐squared tests and Fisher exact tests, and thematic analysis
Dana et al. (2023)^21^	July to September 2020	Curtin University, Western Australia, Australia	*n* = 213 students (70% female, 53% aged 18–24 years, 49% International students). Mean age was 26.07 years.	Cross‐sectional online survey.	Univariate logistic regression and multivariable logistic regression models.
Dharmayani et al. (2024)^19^	October to December 2022.	Macquarie University, NSW, Australia.	*n* = 237 Australian university students (mean age 24.1 ± 7.0 years), 73.2% female, 31.7% International students, 61.6% undergraduate degree.	Cross‐sectional online survey.	Univariate logistic regression modelling and multivariable logistic regression modelling.
Gallegos et al. (2013)^53^	September and October 2009.	Queensland University of Technology, QLD, Australia.	*n* = 810 students (74.4% aged between 17 and 24 years, 75.3% female, 8.3% international students).	Cross‐sectional survey.	Bivariate analysis, Chi square tests and multivariate logistic regression.
Hughes et al. (2011)^54^		Griffith University, QLD, Australia.	*n* = 399 students (80.2% aged ≤24 years, 60.8% female, 29.8% International students, and 79.1% undergraduate).	Cross‐sectional survey.	Chi‐squared test
Jeffrey et al. (2022)^49^	In a first phase between February 2020 and June 2020 (39 mixed students) at the University of Melbourne. 2nd phase From October 2020 to February 2021 (8 international students) at 13 higher educational institutions in Victoria.	First phase: University of Melbourne, Australia Second phase: 13 higher educational institutions in Victoria, Australia.	1st phase: *n* = 87 students 2nd phase: *n* = 48 international students.	Interviews	Not described
Keat et al. (2024)^11^	April to May 2022 and August to September 2022.	Macquarie University, NSW, Australia.	*n* = 29 students (58.6% postgraduate students, 55.2% international students).	Focus groups and semi‐structured interviews.	Six steps of reflexive thematic analysis.
Kent et al. (2025)^51^	July 2022–August 2022	University of Wollongong, NSW, Australia.	*n* = 197 participants (62% female, 72.2% age range 18–24 years, and 63.1% domestic students).	Cross‐sectional online survey.	Logistic regression models.
Kent et al. (2023)^31^	July 2022 and August 2022.	University of Wollongong, NSW, Australia.	*n* = 197 participants (62.5% female, 72.6% aged 18 ‐ 24 years, and 63.5% domestic enrolled students).	Cross‐sectional online survey.	Univariate and multivariate regression models
Kent et al. (2022)^45^	March 2022	University of Tasmania (UTAS) campuses (Hobart, Launceston, and Burnie), and one campus in Rozelle in New South Wales, Australia.	*n* = 1257 students (43% aged 18–24 years, 69% female, 42% enrolled in first year of study, 45% undergraduate courses, 57% studying as ‘on‐campus students’, and 85% domestic students).	Cross‐sectional online survey.	Univariate logistic regression and multivariable logistic regression.
Kent et al. (2025)^46^	March 2022 and March 2024.	University of Tasmania (UTAS) campuses (Hobart, Launceston, and Burnie), and one campus in Rozelle in New South Wales, Australia.	*n* = 1249 students in 2022 and *n* = 1603 in 2024. Aged 18–24 years (44.2%, in 2024 compared to 42.8%, in 2022), females (68.0% in 2024 and 68.9% in 2022), International students increased slightly (15.5% in 2022 to 17.8% in 2024), and first‐year students (52.0% in 2024 to 42.0% in 2022).	Repeated cross‐sectional online surveys.	Univariate logistic regression and multivariate logistic regression.
Ke et al. (2023)^48^	April–June 2019 and follow‐up during the pandemic (Sept‐Oct 2020).	The University of Melbourne, Victoria, Australia.	Wave 1, *n* = 2514 students and Wave 2, *n* = 1250 students were completed the survey.	Longitudinal study.	Multivariable logistic regression.
Lambert et al. (2024)^55^	August 2023 to January 2024.	Eligible participants were medical, nursing, allied health and teaching students in their final year of the degree from 21 universities in Australia or New Zealand.	*n* = 530 responses (65% health professional students, 95.3% domestic student, 83.2% female, 88.3% studying full time, and median age 25 years).	Cross‐sectional online survey.	Chi‐squared tests or Fisher's exact tests were used. Simple thematic analysis.
Micevski et al. (2014)^56^	2012	Deakin University, Victoria, Australia.	*n* = 124 responses (female (*n* = 94), 19–24 years (*n* = 87)).	Cross‐sectional design.	Chi‐square test and multinomial regression analyses.
Mihrshahi et al. (2022)^22^	July and September 202.	Macquarie University, NSW 2109, Australia.	*n* = 105 students (86.3% aged under 30 years, 84.6% female, 61.6% Australian citizens or permanent residents student, and 57.1% were postgraduate students) (including PhD candidates).	Cross‐sectional online survey.	Pearson's Chi‐Square test and the independent samples *t*‐test.
Murray et al. (2021)^32^	March 2020.	University of Tasmania three main Tasmanian campuses (Hobart, Launceston and Cradle Coast) and a campus in the inner‐city suburb of Rozelle in Sydney, New South Wales.	*n* = 1858 respondents (42% aged between 18 and 24 years, 70.3% female, 19.6% International student, and 37% first year of study).	Cross‐sectional online survey.	Chi‐square test with Cramer's V (effect size) and multivariable logistic regression.
Shi et al. (2023)^14^	October 2021 to May 2022.	University of Sydney, Sydney, Australia.	467 students (80.5% domestic students, 74.9% female, 73.0% undergraduates, and 91.2% full‐time students).	Cross‐sectional online survey.	Chi‐square test, Fisher's exact test, the independent sample t test, Mann–Whitney U test, multivariable binary logistic regression model.
Shi et al. (2022)^20^	October 2021 and May 2022.	University of Sydney, NSW, Australia.	*n* = 141 participants (81% female, 74% undergraduate student, 59% lived at the parental home, and 69% employed).	Cross‐sectional survey	Analysis of covariance and the Mann–Whitney U test.
Shi et al. (2023)^47^	March 2020 to April 2022.	University of Sydney, Sydney, New South Wales, Australia.	*n* = 14 participants. The mean (± SD) age was 22.4 (± 2.9) years, female students (*n* = 11), from China (*n* = 8) and from India (*n* = 6).	Qualitative study—semi structured interviews	Interpretative Phenomenological Analysis (IPA) and NVivo (QSR International).
Whatnall et al. (2019)^12^	October 2017 to March 2018.	University of Newcastle, NSW, Australia.	*n* = 366 participants (mean age was 27.3 years, 71.0% female, 85.5% born in Australia, and 72.1% receiving financial support).	Cross‐sectional online survey.	Multivariate logistic regression model.
Whatnall et al. (2020)^52^	September to October 2017.	University of Newcastle, Callaghan, Australia.	*n* = 3077 students (mean ± SD age of participants was 27.1 ± 9.8 years, 69.4% female, 80.9% Australian born, 70.9% undergraduate students, 88.6% domestic students, and 62.0% receiving some financial support).	Cross‐sectional self‐report online survey.	Not discussed

Across 17 studies using validated United States Department of Agriculture (USDA) Household Food Security Survey Module (HFSSM) instruments (6‐, 10‐, or 18‐item), prevalence of food insecurity ranged from 9.0% to 77.9%, with higher estimates typically observed in studies using the six‐item short form and when marginal food security was coded as ‘food insecure’. By instrument: 6‐item (10 studies) 18%–77.9%; 18‐item (5 studies) 9%–48%; 10‐item (2 studies) 46.4%–48%. Two additional studies using short tools (single‐item; USDA community toolkit) reported 46.4%–48%. In studies with shorter length survey tools (one study each used the USDA Community Food Security Assessment Toolkit and the single‐item measure of food insecurity (yes/no)), the prevalence was between 38% and 46.5%.^32^, ^54^ Nine studies reported an overall prevalence of food insecurity, while 10 studies reported the severity according to categories of marginal, moderate food insecurity/low food security and severe food insecurity/very low food security.^9^, ^19^, ^31^, ^32^, ^45^, ^46^ Coding of food insecurity differed between some studies adopting the same measurement tool. For example, in four studies, marginal food insecurity was reported as participants being food insecure,^31^, ^45^, ^46^, ^51^ whereas in eight other studies, marginal food insecurity was coded as food secure.^11^, ^12^, ^14^, ^15^, ^19^, ^20^, ^22^, ^53^

In two of fifteen studies that explored the demographic predictors of food insecurity, international students were reported to be at higher risk of food insecurity.^48^, ^50^ Several studies consistently identified low income and limited financial resources as increasing the likelihood of being food insecure.^50^, ^53^, ^54^ Lack of stable employment and limited job opportunities also significantly increased the risk of food insecurity.^9^, ^20^, ^53^ Living alone was also identified as a risk factor for university students^9^, ^12^, ^14^, ^20^, ^31^, ^49^, ^53^, ^54^, ^56^ and younger students (e.g., 18–24, 25–34 years) generally had higher odds of food insecurity compared to older students.^12^, ^14^, ^32^, ^45^, ^46^ A lack of access to government financial support was a significant contributing factor to food insecurity among international students.^15^ Food insecurity was associated with support‐seeking behaviours in nine studies, while individual coping strategies such as budgeting and buying cheaper food were identified in four studies.^9^, ^11^, ^14^, ^19^ Other behaviours involved accessing free food from charities, food banks, or other organisations (*n* = 4 studies),^21^, ^31^, ^50^, ^53^ receiving support from friends and/or family (*n* = 2),^21^, ^50^ and income generation (*n* = 1)^54^ (Table 2).

**TABLE 2:** Overview of included studies that reported the prevalence, demographic predictors and coping strategies associated with food insecurity in Australian university students.

Author, year, and references	Measurement tool and scoring	Prevalence of food insecurity	Sociodemographic characteristics and related factors	Coping strategies adopted
Bennett et al. (2022)^9^	6 item USDA HFSSM: possible scores range from 1 to 6 with higher scores denoting greater instances of food insecurity.	18% experienced food insecurity (low and very low food security) and 14% were marginally food secure.	Food insecurity was associated with being an international student, unemployed and looking for work (*p* < 0.001), living alone (*p* < 0.001).	Food insecurity was associated with reporting needing to ask family or friends for food, needing to shop at multiple outlets for discounts, the use of emergency food relief, accessing subsidised meals and financial assistance from organisations increased (*p* < 0.002). Shopping for specials or discounts across a number of food outlets.
Brownfield et al. (2023)^15^	6 items USDA HFSSM: food secure (=0) or marginal food insecurity (=1–2) Food insecurity = low food security (=3–5) or very low food security (=6–10).	25.5% food insecure. Marginal food insecurity was included as food secure.	Renting (3.04 times), receiving government benefits (0.65 times), having a disability (0.46 times), and being older (1.19 times) were positively associated with food insecurity.	Not discussed
Waite et al. (2025)^50^	10‐item USDA HFSSM: food secure = high food security (=0) or marginal food insecurity (=1–2) Food insecurity = low food security (=3–5) or very low food security (=6–10).	48% participants experienced food insecurity, but severity not reported.	Participants who reported annual income ≤AUD$18200 per year and the first year after arriving in Australia (67%) and were also more likely to experience food insecurity.	The most common strategies included receiving free food from a charity, food bank or religious organisation (63%), financial management in the form of working extra hours (61%), asking friends or family for money (53%), visiting family or friends to eat food (50%) and borrowing food from friends or family (44%).
Dana et al. (2023)^21^	18 item USDA HFSSM: high food security = food secure (=0), marginal food security (=1–2), low food security (=3–7), and very low food security (=8–18).	48% students experienced food Insecurity, severity of food insecurity not reported. Marginal food insecurity was included as food secure.	The likelihood of experiencing food Insecurity was 9.13 times among international students.	Obtained food support from their friends and/or family, accessed student guild food parcels, and received food hampers from charity organisations.
Dharmayani (2024)^19^	10‐items USDA HFSSM: food secure = high food security (=0), marginally food insecure (= 1), moderately food insecure (= 2–5), and severely food insecure (= 6–10). For analyses, ‘food secure’ (<2) and ‘food insecure’ (≥2).	46.4% were identified as food insecure (29.5% and 16.9% moderately food insecure and severely food insecure). Marginal food insecurity was included as food secure	Food insecure students were significantly more likely to live in larger household size than food secure students.	Affordability and physical access to food were the most frequently re‐ported reasons students chose particular programs. knowledge and skills to cook healthier meals, including seeking consultation with a nutritionist.
Gallegos et al. (2013)^53^	18‐items USDA HFSSM: high food security = food secure (=0), marginal food security (=1–2), low food security (=3–7), and very low food security (=8–18).	25.5% students were food insecure. Marginal food insecurity was included as food secure.	Compared to students who were living at home, those who boarded (2.29 times) or rented (3.40 times) were more likely to experience food insecurity respectively. Students in the lowest tertile for income (1.81 times) was more likely to experience food insecurity than their higher income counterparts. Regarding the source of income, students who participated in part‐time work, obtained income support and received an equity‐based scholarship from the university (3.63 times) were more likely to report being food insecure compared to those who participate in full‐time work.	University‐sponsored food bank or other welfare agencies.
Hughes et al. (2011)^54^	USDA Community Food Security Assessment Toolkit.	46.5% of the student was food insecure without hunger, while 25.3% experienced a more severe form of food insecurity (food insecurity with hunger).	Student food insecurity was significantly associated with those renting, boarding or sharing accommodation, with low incomes or receiving government assistance.	Coping strategies developed by students focused on income generation and austerity measures, included living with parents, working more than 10 h per week outside of university and borrowing money and food.
Jeffrey et al. (2022)^49^	Produced an animated film about student food insecurity, developed a toolkit for universities seeking to address the problem of food insecurity, and organised a workshop on student food insecurity in Australia.	Students experienced food insecurity in relation to other domains of life, such as work and health.	Food insecurity was an abiding concern for many students of all genders, including undergraduates and post‐graduates as well as international and domestic students in Victoria. Students who lived in private or university accommodation appeared to suffer from the most intense forms of food insecurity.	Not discussed
Keat et al. (2024)^11^	6‐Items Short Form USDA HFSSM: food secure = high food security (=0–1), low food security (=2–4), and very low food security (=5–6).	100%—food insecure students purposively sampled. Marginal food insecurity was included as food secure.	The main barriers to accessing healthy foods were related to availability, pricing, and knowledge of healthy foods.	Food relief boxes and grocery Vouchers.
Kent et al. (2024)^51^	6‐items USDA HFSSM: food secure (=0–1), food insecure (≥2).	54.3% food insecure. Marginal food insecurity was included as food insecure.	Not discussed	Not discussed
Kent et al. (2023)^31^	6‐items USDA HFSSM: food secure = high food security (=0), marginally food insecure (=1), moderately food insecure (=2–4), and severely food insecure (=5–6). For analysis food secure (0) or food insecure (≥1).	54% experienced food insecurity (14% mild, 23% moderate and 18% severe food insecurity). Marginal food insecurity was included as food insecure.	Compared with female students, male students were more likely to be food insecure (1.95 times). In comparison with students who lived at home, students who lived on campus (2.5 times) or in a rental/share house (2.5 times) were significantly more likely to be food insecure.	Food‐insecure respondents were more likely to use the campus food pantry (2.3 times) but not the campus community garden.
Kent et al. (2022)^45^	6‐items USDA HFSSM: food secure = high food security (=0), marginal food security (=1), low food security (=2–4), and very low food security (=5–6).	The prevalence of food insecurity was 41.9% comprising 8.2% marginal, 16.5% low, and 17.3% very low food security. Marginal food insecurity was included as food insecure.	Younger aged between 18 and 24 years (2.22 times), non‐binary, first‐year enrolled (3.46 times), on campus (1.33 times), and international students (1.94 times) were at significantly higher risk of food insecurity.	Not discussed
Kent et al. (2025)^46^	6‐items USDA HFSSM: food secure = high food security (=0), marginal food security (=1), moderately food insecure = low food security (=2–4), and severely food insecure = very low food security (=5–6). For analysis food secure (0) or food insecure (≥1).	Food insecurity increased from 42.0% in 2022 to 52.7% in 2024 (moderate food insecurity was slightly higher from 16.6% in 2022 to 17.7% in 2024). Marginal food insecurity was included as food insecure.	Food insecurity increased across all age groups from 2022 to 2024, with the biggest increase reported among the younger age groups: 18–24 years (4.8 times) in 2022 to (5.7 times in 2024), 25–34 years (4.6 times) in 2022 to 2024 (5.9 times), on campus students showing a larger increase in 2022 (1.9 times) to 2024 (2.1 times), and international students' compared to domestic students decreased from 2022 (2.5 times) to 2024 (1.6 times).	Not discussed
Ke et al. (2023)^48^	Financial difficulties were assessed in the Wave 1 survey by two questions ‘In the last 12 months, were there any times that you ran out of food and could not afford to buy more’ and ‘In the last 12 months, were there are any times you could not afford to buy medicine’. Students who answered ‘yes’ to either question was identified as having financial difficulties.	2019 (7.6%), 2020 wave 1 (7.7%), and 2020 wave 2 (7.4%) in financial difficulties.	Students with high social support during the COVID‐19 pandemic were less likely to report major depression (0.15 times) and anxiety (0.28 times) in Wave 2 comparing to peers.	Not discussed
Lambert et al. (2024)^55^	6‐items USDA HFSSM.	77.9% food insecure on placement (10.4% marginal, 32.1% moderate, 27.7% severe food insecurity).	A higher proportion of health students required financial support (*p* = 0.0001). Health students had higher total costs for the recent placement (AUD$1500; IQR, 600–3453) compared to teaching students (AUD$1200; IQR, 600–2757), likely due to longer placement duration (6 weeks for health students).	Not discussed
Micevski et al. (2014)^56^	18 and 10 items USDA HFSSM: high food security = food secure (=0), marginal food security (1–2), low food security (3–7 for students with children, 3–5 for students without children), and very low food security (8–18 for students with children, 6–10 for students without children).	18% reported experiencing food insecurity without hunger and a further 30% experienced the more severe food insecure with hunger.	A lower odd of being food insecure was reported among students living with their family (0.35 times without hunger; 0.29 times with hunger), while a higher odd was found among those receiving government support (2.52 times with hunger).	Not discussed
Mihrshahi et al. (2022)^22^	6‐items USDA HFSSM: food secure (0–1), low food security (2–4) or very low food security (5–6). For analysis food secure (0–1) or food insecure (≥2).	41.9% food insecure (34.3% and 7.6% low food security and very low food security) and psychological distress (52.2%, with high and very high levels). Marginal food insecurity was included as food secure.	International students reporting significantly 9.86 times higher food insecurity.	Not discussed
Murray et al. (2021)^32^	Single‐item measure of food insecurity (yes/no): food security: food secure (students reporting “never”), food insecure (students reporting “rarely, occasionally, often, very often and always”).	38% food insecure (70% female, 80% domestic student, 42% aged 18–24 years) were food insecure.	Food insecurity was positively associated with aged between 25 and 34 years (1.85 times) being an international student (1.99 times).	Not discussed
Shi et al. (2023)^14^	18‐items USDA HFSSM: high food security = food secure (=0), marginal food security (=1–2), low food security (=3–7), and very low food security (=8–18).	14.2% were food insecure (13.3%, 8.4%, 5.8%) were marginal food insecure, low food insecure, and very low food insecure. 13.0% domestic students, 18.7% international students. Marginal food insecurity was included as food secure.	Undergraduate students (2.93 times), first‐year students (2.48 times) and students in the fourth year or above of their degree (3.83 times), International students who experienced living arrangement changes due to the pandemic had an increased risk of food insecurity (3.50 times), and international students (2.02 times) had higher odds of being food insecure.	All food‐insecure international students had experience in purchasing cheaper or processed foods. In food‐insecure students, the percentage of students who obtained food assistance was significantly higher in international students (64.7%) than in domestic students (16.3%; *p* < 0.001).
Shi et al. (2022)^20^	18 and 10‐items USDA HFSSM: high food security = food secure (=0), marginal food security (1–2), low food security (3–7 for students with children, 3–5 for students without children), and very low food security (8–18 for students with children, 6–10 for students without children). For analysis high and marginal food security = the food secure group, low and very low food security = food insecure.	9% food insecure. Marginal food insecurity was included as food secure.	Domestic students who experienced difficulties in finding employment due to the pandemic had a lower score for diet quality than those without such experiences. International students who experienced changes in living arrangements (e.g., moved to shared accommodations) had a higher score for diet quality than those without changes. Domestic students with self‐perceived excellent cooking skills and higher cooking frequency (4 days or more per week) had a higher score for diet quality than those with poorer skills and lower cooking frequency.	Not discussed
Shi et al. (2023)^47^	Not assessed	Not discussed	Not discussed	Not discussed
Whatnall et al. (2019)^12^	6‐items USDA HFSSM: high/marginal food security (=0–1), low food security (=2–4) and very low food security (=5–6).	48.1% food insecure (24.9% low food security and 23.2% very low food security). Marginal food insecurity was included as food secure.	Students living in rental accommodation (2.39 times), undergraduate students (3.50 times), and students enrolled in enabling courses (2.99 times) were more likely to be food insecure.	Not discussed
Whatnall et al. (2020)^52^	Not discussed	22% food insecure.	Not discussed	Not discussed

Abbreviations: HFSSM, Household Food Security Survey Module; USDA, United States Department of Agriculture.

Diet quality was explored in 10 studies, six of which reported poor dietary diversity to be significantly associated with higher odds of food insecurity status.^14^, ^19^, ^20^, ^31^, ^52^, ^53^ The association between food insecurity and physical health outcomes was explored in six studies, five of which reported a significant association between food insecurity and fair/poor self‐reported health, with a consistent trend towards poorer physical health among food‐insecure respondents.^9^, ^14^, ^49^, ^53^, ^54^ In qualitative studies, students described how food insecurity exacerbated their existing health concerns and issues with weight management.^11^, ^14^

The association between food insecurity and mental health outcomes was explored in nine studies, six of which reported a significant association between food insecurity and anxiety,^9^, ^48^ depression,^9^, ^21^, ^48^ and stress.^9^ Other studies examined diagnosed mental illness, showing a higher prevalence of conditions such as psychological distress,^15^, ^22^, ^52^ including feelings of shame, embarrassment, guilt, and stress,^11^ among individuals experiencing food insecurity. Two of the four studies found that food insecurity was significantly associated with poorer academic performance (Table 3).^15^, ^53^

**TABLE 3:** Overview of included studies that reported the health, academic and dietary outcomes associated with food insecurity.

Author, Year and Reference	Physical and mental health	Academic	Diet
Bennett et al. (2022)^9^	Food insecurity was associated with higher anxiety (Marginal: 2 times, Low: 3.5 times, Very low: 7.4 times) and depression (Marginal: 2 times, Low: 3.7 times, Very low: 7.2 times), and stress (as per PSS‐10) (Marginal: 2.2 times, Low: 2.7 times, Very low: 4.2 times). Food insecurity had a negative relationship with well‐being (WHO‐5) (Marginal: −7.8 times, Low: −9.0 times, Low: −10.6 times).	Not collected	Not collected
Brownfield et al. (2023)^15^	Food insecurity (β = 0.37, *p* < 0.0001), renting (β = −0.11, *p* = 0.0072), part‐time study (β = 0.11, *p* = 0.0028), and gender (β = 0.11, *p* = 0.0034), significantly predicted psychological distress scores.	Food insecurity was associated with poorer academic performance and increased psychological distress. Women obtained higher WAMs relative to men, and food insecurity was negatively associated with WAMs. Psychological distress was also inversely associated with WAM.	Not collected
Waite et al. (2025)^50^	Not collected	Not collected	Not collected
Dana et al. (2023)^21^	Depression was 1.62 times significantly positively associated with food insecurity.	Not collected	Not collected
Dharmayani et al. (2024)^19^	Not collected	Not collected	Lower consumption of fruit (<2 serves) (1.81 times), students who reported that they ‘strongly disagree/disagree’ that they have easy access to fruit and vegetables (3.43 times), fruit and vegetables were ‘too expensive’ (4.31 times, and 16.70 times) showed significant associations with higher odds of food insecurity status.
Gallegos et al. (2013)^53^	Students from food insecure households were 2.07 times more likely to report only fair or poor general health due to financial difficulties.	Students from food insecure households were 2.99 times more likely to have deferred their studies due to financial difficulties.	Students who were food insecure were 0.65 times less likely to consume adequate (more than two) serves of fruit per day, and 0.45 times less likely to consume adequate (four or more) serves per day of vegetables.
Hughes et al. (2011)^54^	Students reporting to be food insecure according to the single‐item measure were less likely to rate their overall health as good to very good, when compared with their food secure counterparts (70% vs. 87%).	Not collected	Not collected
Jeffrey et al. (2022)^49^	Prolonged food insecurity led many students feeling anxious or depressed, and some reported physical health issues like lethargy, insomnia, and thyroid problems. Those with existing conditions, such as diabetes or coeliac disease, said food insecurity worsened their health.	Lack of sufficient nutritious food had compromised their capacity to engage successfully with lectures and tutorials and study independently.	Wish to buy cheap food that was quick to prepare, especially ready‐made pizzas, potatoes, rice, and frozen vegetables.
Keat et al. (2024)^11^	Students reported physical effects of food insecurity including weight loss and gain, loss of energy, nutrient deficiencies, head‐ aches, and gastrointestinal issues. Additionally, mental impacts of food insecurity, including feelings of shame, embarrassment, guilt, and stress due to the increased prices of some healthy foods, resulting in frustration.	Not collected	Factors that have influenced their eating habits since becoming a student were also mentioned including time constraints and the availability of foods around campus.
Kent et al. (2024)^51^	Not collected	Not collected	
Kent et al. (2023)^31^	Not collected	Not collected	Food‐insecure students had 3.5 points lower diet scores, with significantly lower vegetable (1.3 points) and fruit (0.8 points) scores, and borderline lower dairy scores.
Kent et al. (2022)^45^	Not collected	Not collected	Not collected
Kent et al. (2025)^46^	Not collected	Not collected	Not collected
Ke et al. (2023)^48^	The prevalence of depression was substantially increase from 22.7% (Wave 1) to 43.8% (Wave 2), and anxiety from 26.5% to 45.7%. Overall, rates were 1.8 to 2 times higher than in Wave 1. Overall, the weighted prevalence of major depression and anxiety were seen to be 1.8 to 2 times higher than in Wave 1.	Not collected	Not collected
Lambert et al. (2024)^55^	Themes of burnout, emotional distress, inability to focus on learning, postponing care of oneself, urgent need for financial support, unanticipated family and other circumstances and worsened societal inequity.	Not collected	Not collected
Micevski et al. (2014)^56^	Not collected	Not collected	Not collected
Mihrshahi et al. (2022)^22^	International students reporting significantly higher psychological distress scores (2.68 times). Food‐insecure students had a significantly higher prevalence of psychological distress (8.07 times) compared with food‐secure students.	Not collected	Not collected
Murray et al. (2021)^32^	Not collected	Not collected	Nearly, half (47%) of food insecure students were dissatisfied or very dissatisfied with the availability of affordable food on campus.
Shi et al. (2023)^14^	Both food‐insecure domestic (1.94 times), and international (2.98 times) students were more likely to experience poor well‐being than their food‐secure peers.	The effects of food security status on class attendance, academic performance, and withdrawal from courses were reflected by domestic students.	Among domestic food‐insecure students, food insecurity was linked to lower cooking confidence and fruit intake. For international students, the provision of meals in accommodation other than their own home (e.g., residential colleges and homestay) were associated with higher odds of food insecurity (4.55 times) than those living in accommodations without provided meals.
Shi et al. (2022)^20^	Better diet quality was also associated with better self‐reported physical health status in domestic students.	Not collected	Food‐insecure students had a poorer diet quality than their food‐secure peers.
Shi et al. (2023)^47^	Weight fluctuations were commonly experienced and craving for traditional foods no longer accessible may negatively impact mental health.	Not collected	International students in Australia consumed more international foods, dairy, and animal proteins due to greater availability but faced challenges accessing vegetables and traditional foods because of limited supply and higher costs.
Whatnall et al. (2019)^12^	Not collected	Not collected	Not collected
Whatnall et al. (2020)^52^	22.5% high and 15.1% very high risk of psychological distress.	Not collected	49.3% were not meeting fruit consumption recommendations and 89.5% were not meeting vegetable consumption recommendations.

**FIGURE 2:**
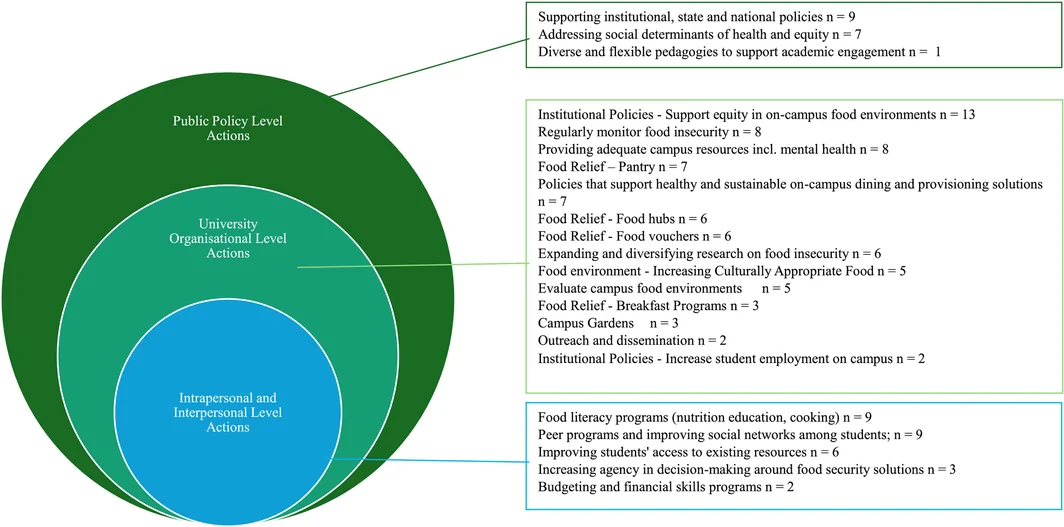
Author recommendations, based on the findings of the systematic review, for addressing student food insecurity.

In each of the cited studies included in this review, author recommendations that resulted from the reported findings have been summarised and grouped according to levels involving public policy, institutional, and individual actions (Figure 2). Most of the recommendations related to activities and strategies that could be implemented at the community level (i.e. university setting). At the policy level, they emphasise addressing social determinants of health and supporting inclusive policies. Institutionally, key recommendations include equitable on‐campus food environments, regular monitoring, comprehensive resource provisioning mental health and food relief services, culturally appropriate dining options, and increased student employment. At the individual level, they emphasise food literacy, peer support, improved access to resources, and financial skills training. These multilevel approaches collectively address both immediate food access needs and underlying structural determinants, providing a framework for sustainable food security interventions within educational institutions.

## DISCUSSION

To our knowledge, this is the first review to synthesise evidence on food insecurity among Australian university students and shows that the prevalence is consistently higher than in the general population. In addition, food insecurity is linked not only to poorer diet quality but also to higher levels of psychological distress and there is emerging evidence of adverse impacts of food insecurity on academic performance. While no peer‐reviewed evaluations of interventions to address food insecurity were identified in the Australian context, many of the included studies offered recommendations for potential solutions, providing a starting point for future action and research.

Across the 23 included studies, the prevalence of food insecurity among Australian students ranged widely from 9% to 77.9%, generally exceeding estimates for the general Australian population.^57^ For example, in 2023, food insecurity affected approximately 13% of Australian adults.^57^ The variation between studies may stem, in part, from differences in measurement tools and cut‐offs, particularly related to the inconsistent categorisation of marginal food insecurity. These divergent approaches may bias prevalence estimates upward or downward and limit comparability across studies. Standardising measurement and reporting, particularly for marginal food insecurity, would strengthen future research and policy translation. Further, research has shown that university students may interpret food security questions differently than the general population, potentially leading to misclassification bias.^58^ This misclassification may distort the relationship between food insecurity and outcomes, indicating a need to validate and adapt survey tools specifically for Australian university students from diverse backgrounds.^58^, ^59^, ^60^ To address this, cognitive testing should be undertaken to validate and refine adaptations of the HFSSM for use among diverse Australian university students.

The review highlights financial strain as the fundamental driver of food insecurity among students, exacerbated by multiple structural and personal factors. Income support payments such as Austudy and Youth Allowance have been described as inadequate, falling below the poverty line. Postgraduate students are often ineligible, and while universities offer stipends, these are competitive and not means‐tested.^61^ As a result, many students work alongside their studies, sometimes in informal or cash‐in‐hand jobs. Employment opportunities are often casual, low paid, or incompatible with study demands, producing a paradox where students are forced to balance insufficient part‐time wages with the academic risks of full‐time work, making students particularly vulnerable to food insecurity.^62^ This employment dilemma is further complicated by the reality that even when students secure work, their wages have remained stagnant over recent years while living costs have escalated.^63^, ^64^

Consistent with studies from the USA^65^ and Canada,^66^ this review demonstrates that younger students (18–24 years) face significantly higher odds of food insecurity compared to older students. This heightened vulnerability relates to younger students having more limited financial resources and less established support networks compared to middle‐aged students.^38^ The transition to independent living, combined with inexperience in financial management, creates challenges for this demographic. International students are another particularly vulnerable group, facing substantially greater food insecurity risk due to multiple compounding factors that result in dietary changes, including poor food literacy especially in cooking and grocery shopping skills, limited access to traditional foods, and reduced financial capacity.^67^ Challenges experienced by international students extend beyond financial constraints to include cultural adaptation difficulties, inability to access preferred foods, and social isolation that compounds food security risks.^14^ The lack of access to government financial support payments represents a significant contributing factor to food insecurity among this population,^68^, ^69^ suggesting that university students require enhanced financial support to prevent food insecurity, as supported by approaches in America.^3^ Finally, living independently whether on campus, in rental housing, or in share accommodation also increases vulnerability, compared with students living with family who benefit from shared financial resources, which is consistent with other studies conducted in USA students.^70^, ^71^, ^72^ The financial burden of independent living, particularly in major capital cities like Melbourne and Sydney where living expenses are high, creates substantial budgetary pressure.^73^ Students living at home benefit from reduced expenses and often receive direct parental financial support, creating protective buffers against food insecurity.^74^ In recent years, the strong demand and slowing supply of Australia's rental market has worsened rental affordability and increased financial stress for some renters including university students, who may struggle to cover basic living expenses. A significant portion of their income goes towards housing costs, potentially leaving little for essentials like food, though this relationship requires further research.^75^, ^76^

Our review showed that food‐insecure students were less likely to meet fruit and vegetable recommendations and more likely to rely on cheaper, energy‐dense foods.^74^, ^77^, ^78^ Qualitative studies described coping strategies such as skipping meals, choosing fast foods when constrained by time and money, or, in severe cases, going entire days without eating.^79^ Extending this, the literature also demonstrated strong links between food insecurity and mental health which may be related to worrying about food adequacy. A stepwise relationship was observed between severity of food insecurity and levels of stress, anxiety, and depression.^80^, ^81^, ^82^, ^83^ Qualitative evidence reinforced these findings, with students describing shame, stigma, and helplessness associated with inadequate food access. Importantly, the relationship appears bidirectional, with poor mental health also heightening vulnerability to food insecurity.^29^, ^66^, ^83^, ^84^, ^85^, ^86^, ^87^ Fewer studies examined physical health, but those that did reported poorer general health, fatigue, weight fluctuations, and exacerbation of chronic conditions such as diabetes among food‐insecure students. Although largely self‐reported, these outcomes align with broader research linking food insecurity to compromised physical health.^88^, ^89^ More longitudinal, objective research is needed to confirm long‐term health impacts in this population.

Although few studies (*n* = 4) explicitly examined academic outcomes, half found associations between food insecurity and compromised academic performance.^14^, ^15^, ^49^, ^53^ Reported impacts included reduced concentration, absenteeism, fatigue, and in severe cases, course deferral or withdrawal. Food insecurity therefore represents a barrier not only to health and wellbeing but also impacts educational attainment and career prospects.^82^, ^90^, ^91^, ^92^ The evidence suggests that persistent food insecurity may increase dropout risk, with long‐term implications for both individuals and society, warranting further attention.^93^, ^94^

Although our review did not identify peer‐reviewed evaluations of interventions in the Australian higher education sector, many studies included recommendations for addressing food insecurity. Common suggestions included improving the adequacy and accessibility of student income support, increasing affordable housing options, enhancing on‐campus employment opportunities, and normalising help‐seeking to reduce stigma. Universities were also encouraged to partner with government and community organisations to co‐design sustainable responses that go beyond food pantries or vouchers. These recommendations provide useful direction,^3^, ^12^, ^53^ but rigorous evaluation is still needed to determine their effectiveness and scalability in the Australian context.

This review was strengthened by a registered protocol, a comprehensive search across eight databases, and independent quality assessment. However, study heterogeneity in measurement tools and cut‐offs limited our ability to synthesise food insecurity prevalence estimates. Most included studies were cross‐sectional and relied on self‐reported data, restricting causal inference and raising the possibility of reporting bias. While many studies used established tools such as the USDA HFSSM, few explicitly addressed their validation in Australian or student populations, raising questions about cultural and contextual appropriateness. Conducting validation studies using these tools, such as through cognitive interviewing, in future is warranted. The literature also remains fragmented, with limited longitudinal studies exploring causal pathways between food insecurity, health, and academic outcomes. Further research should also evaluate the long‐term effectiveness of institutional and policy interventions and explore the complex intersections between food insecurity, housing affordability, employment, and mental health in different geographic contexts.

Food insecurity among Australian university students is widespread and persistent, with a consistently higher prevalence than in the general population. Its impacts extend across diet, mental and physical health, and academic performance, positioning it as both a public health concern and an educational equity issue. Current institutional responses provide short‐term relief but fail to address systemic drivers such as inadequate income support, housing affordability, and unstable employment. Sustainable solutions require coordinated, evidence‐based approaches involving universities, government, and community partners. Moving beyond emergency relief towards structural reform is essential to ensure that all students can participate fully in higher education without the burden of food insecurity.

## AUTHOR CONTRIBUTIONS

*Conceptualisation*: SDS, KC, KK. *Searching*: SDS. *Screening*: SDS, KK. *Synthesis*: all authors. *Writing first draft*: SDS, KK. *Writing subsequent drafts*: all authors.

## CONFLICT OF INTEREST STATEMENT

The authors report no declarations of interest. The authors alone are responsible for the content and writing of the article.

## Supporting information


**Table S1.** Search framework (PICOS) and example terms.

**Data S1:** Supporting Information.

## Data Availability

The data that support the findings of this study are available on request from the corresponding author. The data are not publicly available due to privacy or ethical restrictions.
