# Supplementary material for: Food insecurity among university students in Australia: A systematic review of prevalence, predictors, health and academic outcomes
Source: Nutr Diet. 2026 Jun 4;83(4):407–28. doi: 10.1111/1747-0080.70082 (PMC13575728; doi:10.1111/1747-0080.70082)
Supplement: Supplementary file 1 [file NDI-83-407-s001.docx]

**Supplementary Table 1. Search framework (PICOS) and example terms**

| Parameter | Criterion |
| --- | --- |
| Population | Human subjects who were students at an institution of higher education (search terms: "university students" OR "tertiary students" OR "undergraduates" OR graduates OR postgraduates OR "placement students" OR "Higher Degree Research Students" OR HDR OR "research students" OR "TAFE students" OR "domestic students" OR "local students" OR "international students" OR "mature age students") |
| Exposure | Food insecurity (search terms: “food insecure*” OR “food security*” “food availability*” “food insufficiency*” “food sufficient*” OR “food access” food AND security OR scarcity OR insufficient* OR sufficient OR lack OR deprivation OR short* OR nutrition* OR bank OR pantry OR relief OR diet OR hunger OR "skipping meals" OR supermarket OR grocer*) |
| Country | Australia (search terms: Australia OR Australian OR Australians OR "Northern Territory" OR Tasmania OR Queensland OR Victoria OR NSW OR "New South Wales") |
| Outcome/Impact | Food insecurity (search terms: determinants OR impact* OR intervention* OR outcome OR evidence OR cause OR effect OR affect OR contribute* OR factor OR prevalence OR barrier OR result OR survey OR question* OR data) |
| Study design | Peer-reviewed human studies of any design |
